# The human geography of Twitter: Quantifying regional identity and inter-region communication in England and Wales

**DOI:** 10.1371/journal.pone.0214466

**Published:** 2019-04-15

**Authors:** Rudy Arthur, Hywel T. P. Williams

**Affiliations:** Department of Computer Science, CEMPS, University of Exeter, Laver Building, North Park Road, Exeter, EX4 4QE, United Kingdom; University of Southern California, UNITED STATES

## Abstract

Given the centrality of regions in social movements, politics and public administration, here we aim to quantitatively study regional identity, cross-region communication and sentiment. This paper presents a new methodology to study social interaction within and between social-geographic regions, and then applies the methodology to a case study of England and Wales. We use a social network, built from geo-located Twitter data, to identify contiguous geographical regions with a shared social identity and then investigate patterns of communication within and between them. In contrast to other approaches (e.g. using phone call data records or online friendship networks), use of Twitter data provides message contents as well as social connections. This allows us to investigate not only the volume of communication between locations, but also the sentiment and vocabulary used in the messages. For example, our case study shows: a significant dialect difference between England and Wales; that regions tend to be more positive about themselves than about others, with the South being more ‘self-regarding’ than the North; and that people talk politics much more between regions than within. This study demonstrates how social media can be used to quantify regional identity and inter-region communications and sentiment, exposing these previously hard-to-observe geographic concepts to analysis.

## Introduction

Studies of human social interaction using phone call data and online social networks [1–7] have found that, contrary to some expectations [8], geography is alive and well. Despite digital technology decoupling distance and difficulty of communication, spatial proximity remains one of the key factors in determining who communicates with whom. Regions determined from records of telephone communication closely reflect traditional regional and local identities [1]. This has been confirmed for numerous countries [2] and for various different forms of electronic interaction [3].

The notion of a region therefore has much more than a purely bureaucratic meaning. Discussions of regional identity pervade social theory [9, 10], and many stereotypes, sporting rivalries and political differences occur at the regional level. In this paper we will study the regions of England and Wales, which is of particular relevance at a time when British national identity is being challenged by Brexit, regional devolution, and the economic disparity between North and South. However given that national and international policies are often implemented at the regional level (e.g. the European Union cohesion policy see http://ec.europa.eu/regional_policy/en/faq. Accessed June 2018), questions of regional identity have wider geo-political relevance. Given that geographic regions are so fundamental, our question is: how can we quantitatively study ideas like ‘regional identity’, ‘regional rivalry’ or the ‘cultural dominance’ of regions?

We begin with the observation that online social networks tend to have similar properties to offline, spatially embedded, social networks. In fact spatial structure in communication networks is robust enough to have instrumental value. Much recent research, especially on social media, has focused on exploiting the strong spatial correlations that exist in friendship networks to infer the locations of users (e.g. [11–13]). Other work linking social networks to geography has, for example, attempted to determine the amount of commerce in a given area [14] or the location of a city’s ‘heart’ [15]. This field of network geography has mostly focused on the network’s topology and how this influences interaction and accessibility [16]. It is our aim to move beyond this by studying a social network where the links carry much richer metadata.

We analyse a social network of interactions on Twitter. This social network is constructed from ‘mention’ interactions, in which one user explicitly mentions or replies to one or more other users, and thus it has a few interesting properties. Firstly, connections are intrinsically directional. Alice can mention Bob on Twitter without Bob’s permission, and Bob does not have to reciprocate. This allows for asymmetries in communication, so (at the network level) some regions can be the target of more mentions than others. Secondly, and crucially, unlike either phone call networks (where the call content is unknown) or friend/follower networks (which do not imply communication) here we have both the directed link between users *and* the content of the message.

The plan of the paper is as follows. We first demonstrate that user communities, identified algorithmically from Twitter mentions, are geographically contiguous and loosely correspond to our expectations, based on administrative boundaries and ‘folk’ conceptions of British regions. This approach makes no *a priori* assumptions about the number, location or boundaries of different regions, and is independent of administrative demarcations that may or may not reflect real regional identities. Next we use these emergent regions as the subjects of a comparative study of intra- and inter-region communication. We will compare the vocabulary and topics used by members of a region when speaking to each other compared with those used to speak to ‘outsiders’. We will then look at the volume and sentiment of messages sent within and between regions. Thus we can ask questions like “What does the South-West say to North Yorkshire?” and answer them in a concrete way e.g. “They talk about sport, and the sentiment of the communication is slightly more negative than average”.

## Materials and methods

Our dataset of tweets from all of England and Wales (defined by a bounding box with lower left longitude and latitude (-5.8,49.9) and upper right (-1.2,55.9)) was obtained from four separate collections, one for the South-West (which was ongoing from previous work [17]) and the other three chosen to sample an area containing ∼15 million people each. This collection lasted from 01/10/2017 to 22/03/2018. All data was gathered in compliance with all applicable Twitter API policies and terms of use (see https://developer.twitter.com/en/developer-terms/policy). In accordance with Twitter’s Developer Policy (see https://developer.twitter.com/en/developer-terms/agreement) we use only a single API key which limits us to 1% of the total global Twitter stream. As we collect a narrow subset of all Tweets, namely geo-tagged Tweets from England and Wales, we do not expect this restriction to bias our sampling. Only a very small percentage of tweets are geo-tagged, and the UK is only a small part of the global Twitter user base. Thus we do not expect that these rate limits will affect our sampling and we see no evidence of this in our data.

All of our tweets have geographical information attached as GPS co-ordinates or ‘place-tags’. Previous work [17] has shown that GPS tagged posts are predominantly shares from other social media platforms or automated accounts, while place-tagged tweets represent direct human interactions on Twitter. Thus we exclusively use place tags for location and discard GPS-tagged tweets. We locate users by assigning them to grid tiles proportionally to the frequency of their tweeting within the tile. For example, we can have 0.5 of a user in tile 1 and 0.5 in tile 2 if that user tweets equally often from 1 and 2. This is preferred to using the user location field which is often blank, doesn’t contain location information or is too vague (e.g. ‘England’). We end up with 4513957 useful tweets authored by users in England and Wales and which mention users in England or Wales (excluding self mentions). All of our analyses are performed with this set.

## Identifying regions with tweets

Previous research has shown that geographical regions can be recovered from communications data using network analysis. In this section, we show that this approach can be successful with data from Twitter. In our set of ∼4.5 million tweets there are ∼375, 000 unique users who mention another user in the target area. The mention network is constructed by treating each grid tile as a node and then adding an edge, *e*_*ab*_, between every pair of tiles *a* and *b* when a user in tile *a* mentions a user in tile *b*. When all tweets are considered *e*_*ab*_ is the total number of mentions by users in *a* of users in *b*. Edges are directed and have weight equal to the number of mentions sent from tile *a* to tile *b*. Self-edges (i.e. *a* = *b*) are allowed.

The Louvain method [18] is used to find communities within the resulting network; this method of community detection is robust, fast and automatically determines the best (modularity maximising) number of communities. However, this method is intended to work on undirected graphs. To turn the directed mention network into an undirected graph we set the edge weight between every pair of tiles as the total number of tweets sent in either direction (*e*_*ab*_ + *e*_*ba*_), ignoring self-edges. We run the Louvain algorithm with 100 random restarts (to sample multiple local maxima) and choose the community partition with highest modularity from the set of 100 outcomes.

**Fig 1:**
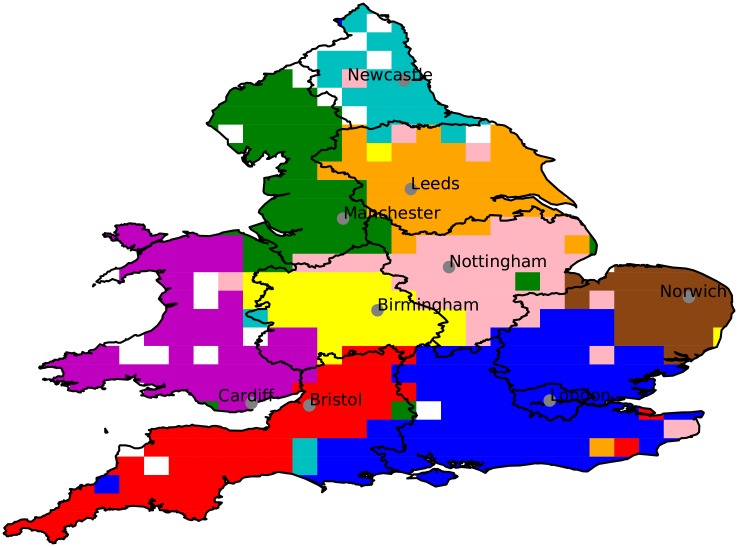
Communities in England and Wales determined from Twitter mentions. The Louvain algorithm suggests 9 communities is optimal for this grid resolution. The largest city in each community is labelled. White space means no tweets were recorded. The regions identified correspond roughly with England and Wales’ administrative regions (shown in grey on the map) South-West (Bristol/red), Wales (Cardiff/magenta), West-Midlands (Birmingham/yellow), East-Midlands (Nottingham/pink), East Anglia (Norwich/brown), Yorkshire and the Humber (Leeds/orange), North-West (Manchester/green) and North-East (Newcastle/cyan). London subsumes two administrative regions: London and South-East.

Fig 1 shows the resulting regional communities, presented as a spatial grid with each tile coloured by its community label. These communities were found (with modularity *Q* = 0.209) using a network constructed using a 30 × 30 grid.

**Fig 2:**
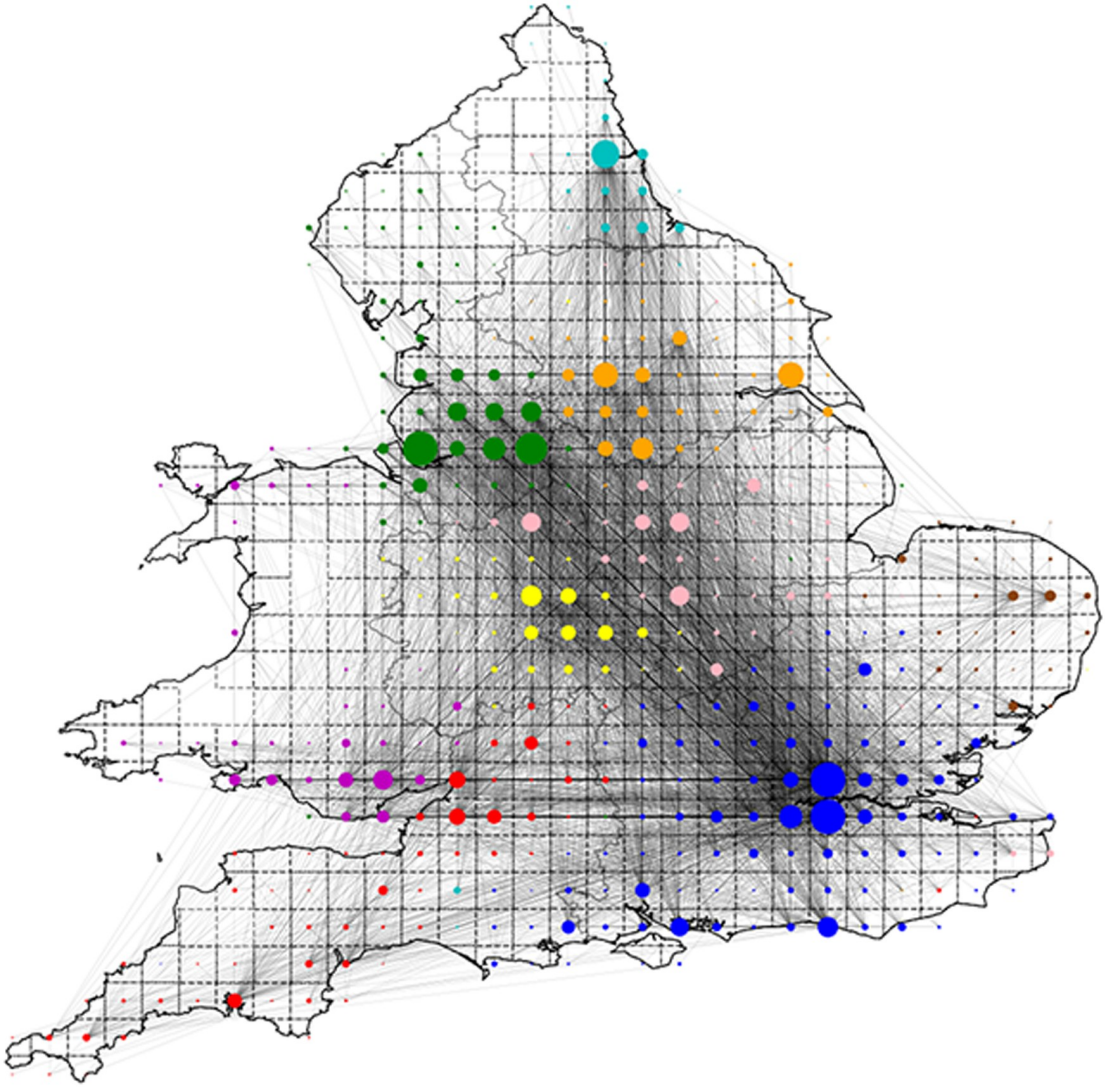
The undirected network of Twitter mentions in England and Wales aggregated on a 30 × 30 grid. The node sizes correspond to the number of tweets sent within a tile i.e. the size of the self edge *e*_*aa*_. Colours correspond to the community allocation discussed in the text. Only connections where *e*_*ab*_ + *e*_*ba*_ > 100 are shown. The map is reflective of population density, showing large numbers of tweets originating in the south-east and north-west. We also see significant communication flow between the regions, supporting our assertion that this data set can be used to study inter-region communication and sentiment.

Examining the map shown in Fig 1, there is a striking geographical coherence to the communities, with 9 contiguous regions easily identified. There are some ‘outliers’, tiles belonging to a community different than their neighbours. These outliers typically have low populations, and hence low numbers of Twitter users and small edge weights, so their assignment has very little effect on the total modularity. Overall the communities reflect ‘folk’ preconceptions of where the regions of the UK should be, have a reasonable correspondence to administrative regions, and also agree with previous work using phone call networks [1]. The main difference occurs in the London region, where London, the South-East and part of East Anglia are incorporated into one region. This area comprises (broadly) the extent of London’s ‘commuter belt’ and is likely an effect of London’s enormous economic and cultural influence. Henceforth will we label each region by its largest city, for ease of reference and since, by examination of Fig 2, most of the communication volume originates from the largest city in a region.

The communication network is shown in Fig 2. We require a non-zero number of internal mentions, i.e. *e*_*aa*_ > 0, in each tile, this *ad hoc* condition removes very sparsely populated tiles which can be assigned to any community without significantly affecting the modularity score. We are left with *N* = 454 nodes/tiles in the graph. The associated undirected mention network contained 65934 edges (i.e. density of 0.641) with mean node degree 31.4 and mean weighted node degree 19808.7. The network has no isolates i.e. it is equal to its giant component. For robustness checks, please see the supporting information in S1 File, where we study the effect the grid resolution has on the detected communities as well as the stability of the communities over time.

## Comparison of vocabulary

Given that people are more likely to be ‘friends’ with people living nearby [11], the types and topics of communications within and between communities may be different. The field of topic modelling, in general and for Twitter, has a large literature (e.g. [19–21]). Other research has studied dialect differences on Twitter, particularly in the USA [22–24]. Here we use a simple approach to compare the words and topics used in intra- and inter-community communication.

We first create a lexicon *W* containing all distinct case-insensitive words (*n* = 556466) from all tweets. We removed user names prefaced by an ‘@’ symbol, URLs, special characters (e.g. emojis), as well as the ‘#’ symbol prefacing hashtags, though we kept the hashtag itself. *W* defines our word-vector space within which we construct TF-IDF vectors (we use the Python sklearn package: https://scikit-learn.org/stable/modules/generated/sklearn.feature_extraction.text.TfidfTransformer.html. Last accessed: 11th March 2019).

**Fig 3:**
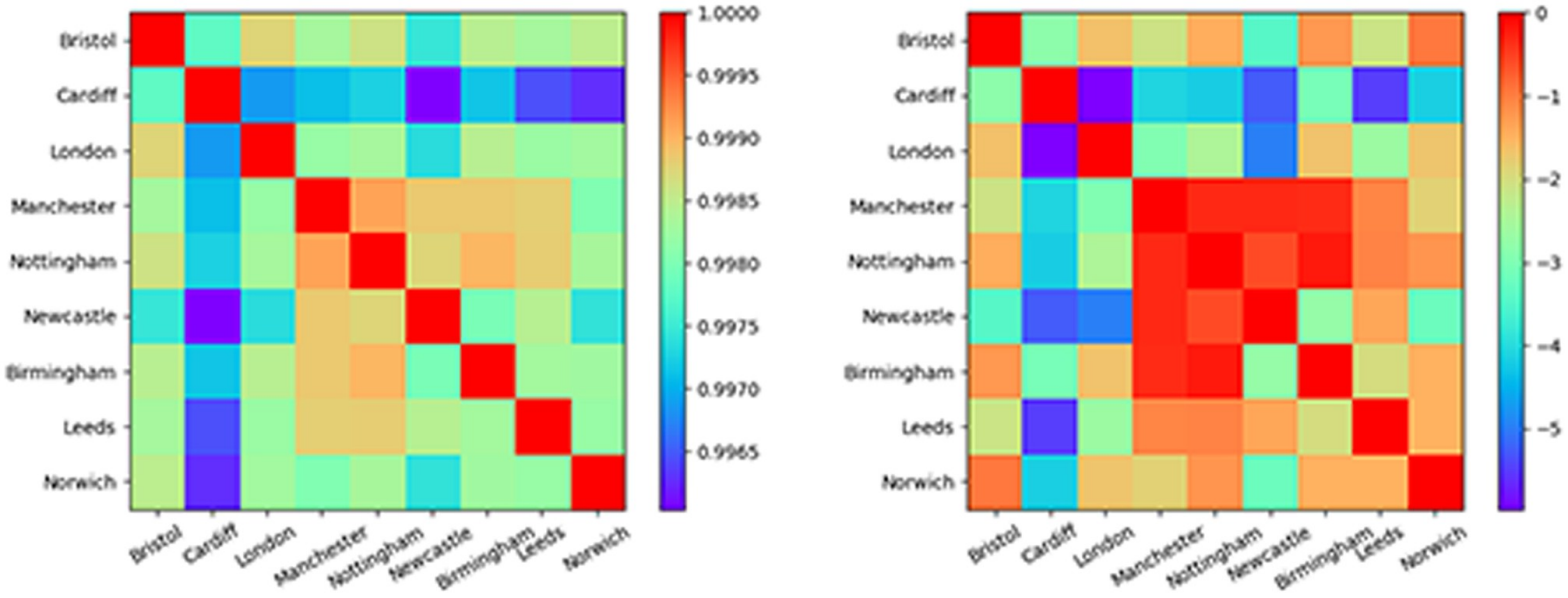
Left: Cosine similarity for word vectors corresponding to each community’s lexicon. This metric equals 1 for identical vectors. Wales is notably less similar to all other regions while the more northern regions are more similar to each other than the southern ones. Right: Z-score of cosine similarity, mean and standard deviation calculated using randomised communities.

The vector ${\vec{w}}_{i}$, represents the word sets obtained from tweets originating in each region. We use cosine-similarity, $\frac{{\vec{w}}_{i}\cdot {\vec{w}}_{j}}{∥{\vec{w}}_{i}∥∥{\vec{w}}_{j}∥}$, to measure the similarity between regional vocabulary. The left panel of Fig 3 shows that all regions are quite similar to each other (similarity greater than 0.99). Cardiff is the most dissimilar, and there is a suggestion that the ‘northern’ regions (Birmingham, Nottingham, Leeds, Manchester and Newcastle) are more similar to each other than to the southern regions (Bristol, London and Norwich).

To investigate the statistical robustness of our findings, we created a resampling distribution for expected similarity. Each value in this distribution was created from a single resampling of the grid cells making up each region; that is, in each instance we kept the number of grid cells in each region fixed but randomly re-selected grid cells to form the region from the entire map. Equivalently, this can be thought of as keeping the regions fixed but shuffling the grid tiles making up the map. Similarity scores were computed for each resampling instance and 100 instances were aggregated to form a distribution for expected similarity. We show the z-scores for the observed similarities with respect to this expectation in the right hand plot of Fig 3. This analysis shows that the vocabulary difference between Wales and England is significant and that Newcastle has a vocabulary that is significantly different from the southern regions London, Bristol and Norwich.

To further investigate dialect we calculate the TF-IDF (term frequency-inverse document frequency) score. TF-IDF assigns high scores to words that differentiate documents within a corpus. We create 9 ‘documents’ by aggregating the tweets originating from each region. The top TF-IDF words in Cardiff are mainly local place names or words in the Welsh language. Other regions’ highest TF-IDF words are mainly local place names or sports clubs (see Text A in S1 File). This method successfully detects the Welsh language, which combined with the cosine similarity suggests a dialect difference between between Welsh and English tweets.

### Local, regional and national communication

One novel aspect of our methodology is that Twitter data includes the content of messages as well as the network structure through which the messages were transmitted. In this section, we explore whether the topics of communication vary at different scales, e.g. local, regional or national. Specifically, we compare how ‘locals’ communicate with each other to how they communicate with ‘outsiders’.

We divide tweets originating from a region into two categories: tweets sent within the region and tweets sent to other regions. Let $f{\left(w\right)}_{i}^{loc}$ denote word frequencies in tweets sent within community *i* and $f{\left(w\right)}_{i}^{out}$ denote word frequencies in tweets sent from community *i* to any other community. Since word frequencies are affected by the total size of a corpus, making comparison of frequencies between different corpora problematic, we use a rank-based approach to normalise the frequencies and enable fair comparison between corpora. We assign each word a frequency rank *r*(*w*) from most common (*r*(*w*) = 1) to least, so $f{\left(w\right)}_{i}^{loc}\to r{\left(w\right)}_{i}^{loc}$ and $f{\left(w\right)}_{i}^{out}\to r{\left(w\right)}_{i}^{out}$. Looking at the rank differences: $\Delta r_{i}=r{\left(w\right)}_{i}^{loc}-r{\left(w\right)}_{i}^{out}$ then gives us a rough indication of how the vocabulary varies in tweets sent within regions compared to those sent between regions. Positive rank differences indicate a word is more common in inter-community messages and negative rank differences indicate the word is more common in intra-community messages. In order to avoid distraction by rare words with spurious large rank differences, we restrict our analyses to words with frequency per tweet (calculated separately in each region and for *loc* or *out*) greater than 0.1% for every word considered. We can look at pairs of regions in the same way. We rank the words used to communicate between communities *i* and *j*, *r*(*w*)_*ij*_, and the words used to communicate between *i* and all other communities (excluding itself and *j*), *r*(*w*)_*i*,*k*∉{*i*,*j*}_, and look at Δ*r*_*ij*_ = *r*(*w*)_*i*,*k*∉{*i*,*j*}_ − *r*(*w*)_*ij*_ to see what words are characteristic of communication between *i* and *j* specifically. In practice, since the sets of words that are compared originate from the same set of users (e.g. comparing words used by users from the “Norwich” region in tweets directed within the region vs tweets directed outwards from the region) the vocabulary size is typically very similar. This avoids potential issues with different sized word sets, which might otherwise distort frequency rankings.

**Fig 4:**
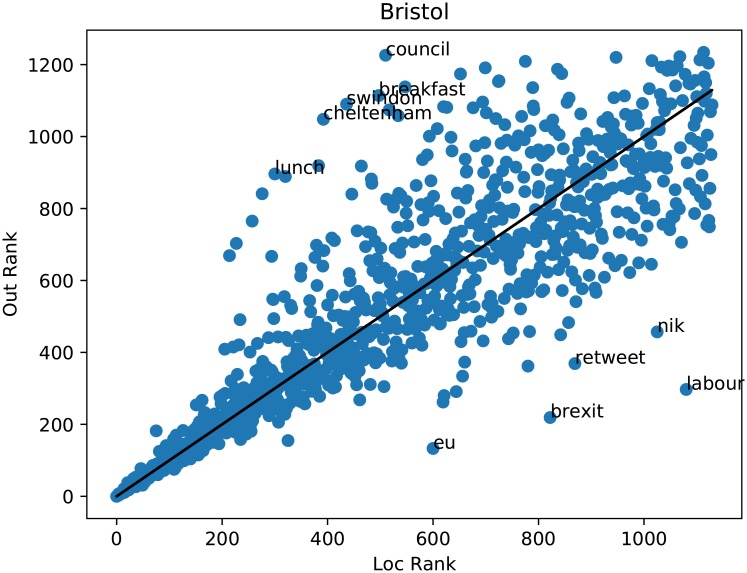
Loc rank versus out rank for Bristol region. Words with largest magnitude rank difference are indicated. Intra-region words include local place names while inter-region words refer to national politics.

Fig 4 and Table 1 show that intra-community words (negative rank difference) primarily refer to local issues like sports (pigeonswoop, villa, mufc) and places in the region (wigan, bradford, chester) similar to the high ranking TF-IDF words. Inter-community words primarily refer to national issues (brexit, eu, nhs, tory). Table 2 shows an example for two neighbouring southern regions. Sport shows up again in pairwise communication, as it does in local communication, but not nationally—indicating that sporting rivalries are playing out on a regional level, as one might expect.

**Table 1:** Top and bottom five rank differences, Δ*r*_*i*_, for 4 most populous regions.

London	Manchester	Birmingham	Leeds
liverpool(613)	brexit(694)	brexit(803)	brexit(682)
sleep(369)	government(612)	disabled(663)	eu(594)
trending(337)	tory(572)	extremely(654)	leaving(570)
ff(316)	nhs(506)	inclusion(654)	nhs(539)
topic(304)	eu(493)	wwfc(518)	ref(520)
co(-396)	wigan(-475)	thursday(-491)	pop(-497)
brighton(-423)	chester(-504)	pigeonswoop(-504)	south(-504)
lunch(-449)	lunch(-515)	villa(-557)	thursday(-512)
awards(-472)	gt(-564)	art(-692)	council(-564)
greater(-818)	mufc(-606)	blues(-700)	bradford(-586)

See Table A in S1 File for the full list.

**Table 2:** Top and bottom five rank differences, Δ*r*_*ij*_, for Bristol to Cardiff and vice versa.

Rank difference	Cardiff to Bristol
30622	busygettingbetter
22063	anglowelshcup
21903	younglivesvscancer
17430	bigwednesdayshow
14548	wenurses
Rank difference	Bristol to Cardiff
19869	tywydd
20289	scarlets
29627	tandyout
40499	tandy
40727	goalscorers

Most of these terms are popular hashtags. Cardiff is talking to Bristol about health related issues, a radio show and a sporting event. Bristol is talking to Cardiff about sports and ‘tywydd’, Welsh for ‘weather’.

## Communication flow between regions

A reasonable hypothesis is that the volume of communication between regions will depend on their geographical proximity and the size of their populations. In this section, we explore this idea by measuring the pairwise volume of communication between regions.

**Fig 5:**
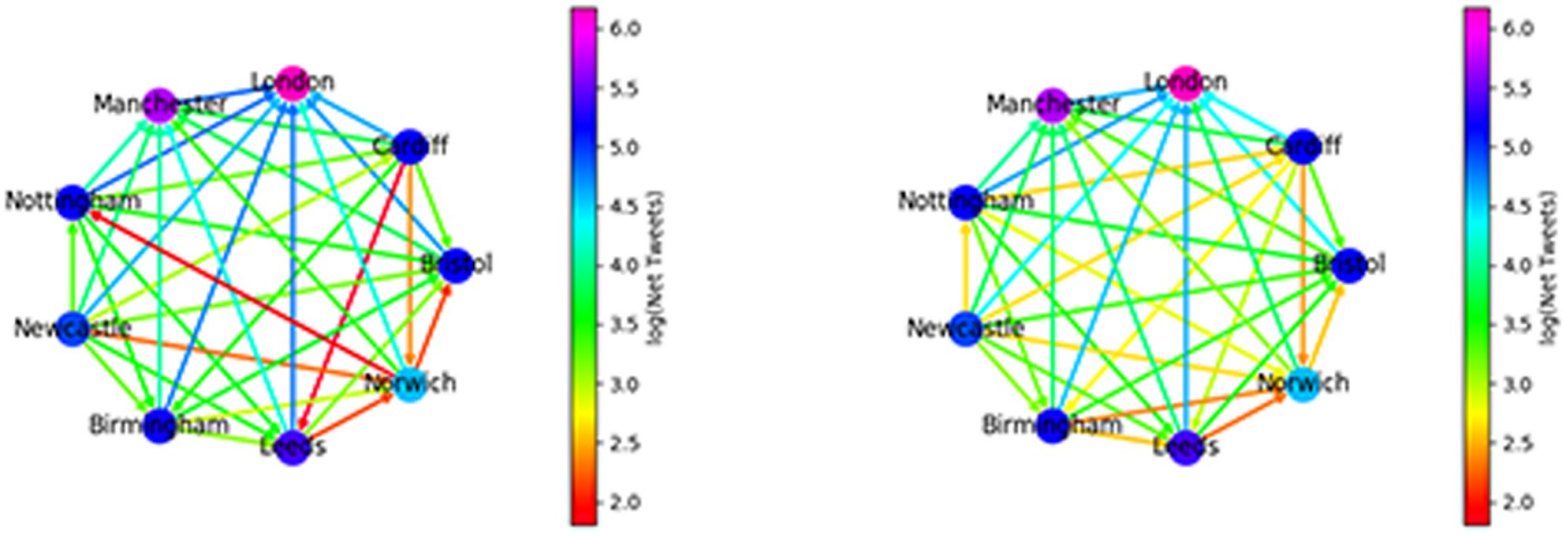
Left: Number of mentions sent between UK regions. Arrows show the direction of flow e.g. Manchester mentions London more than London mentions Manchester. Node colour shows number of mentions sent within a community. Right: The flow of mentions computed via the null model, node colours same as left image.

Now that we have an assignment of each tile to a community, based on the undirected network, we form a directed network induced by the assignment of communities in Fig 1. We want to know about the net flow of mentions i.e. Does London mention Manchester more than vice-versa? Let there be *N* communities and let *m*_*ij*_ be the number of mentions of (users in) community *j* by (users in) community *i*. If *m*_*ij*_ > *m*_*ji*_ we draw a directed edge from *i* to *j* with weight *m*_*ij*_ − *m*_*ji*_. If *m*_*ij*_ < *m*_*ji*_ the edge goes from *j* to *i* with weight *m*_*ji*_ − *m*_*ij*_. Thus our arrows always point towards the region which is mentioned more, weighted by the net difference in communication volume. We show this network in Fig 5 (left).

We see that London (the most populous region) is always mentioned more by the other regions than vice versa. This is perhaps expected, as tweets referencing politics are likely to be directed towards the capital. Manchester (containing the second largest city) is mentioned more by all but London. In light of previous work [17] showing that high population density leads to a super-linear increase in the amount of Twitter activity, this is perhaps not surprising; London and Manchester are very densely populated regions, so contain a lot of users and hence present a large ‘target’ for other regions. However population is not a perfect predictor, contrast Newcastle (always in deficit of mentions) with Norwich. Despite Newcastle having a larger population (∼ 2.7 million versus ∼ 1.6 million, see Table 3.) it is mentioned much less than Norwich, perhaps an indication that its geographic isolation (it is far from the large population centres in the North-West and South-East) is leading to some social isolation.

**Table 3:** Regional populations (using our discovered regions) and ratio of number of incoming mentions to number of outgoing mentions.

Region	Population	$\frac{\sum_{j\neq i}m_{j}^{in}}{\sum_{j\neq i}m_{j}^{out}}$
London	22650335	1.629
Manchester	7733168	0.981
Bristol	4623903	0.838
Leeds	5571395	0.774
Birmingham	5038212	0.746
Norwich	1561239	0.733
Nottingham	5493704	0.690
Cardiff	2818706	0.689
Newcastle	2744728	0.638

Data from UK Census https://www.ons.gov.uk/peoplepopulationandcommunity/populationandmigration/populationestimates (Accessed April 2018).

To see which communities talk more or less than expected we establish a null model by cutting all the outgoing edges and rewiring them randomly, while keeping the in- and out-degree of every node fixed, to account for the relative size and activity of each region. This null model generates the expected pattern of inter-region communication based on Twitter activity in each region, assuming no bias in inter-regional communication. We do not redirect self-edges since the communities are, roughly, chosen to maximise self interaction by the Louvain algorithm. Comparison to a null model which randomly reassigns self-edges will thus show that the observed graph has more self interaction, by construction. It is more informative to focus on inter-community communication only. A community *i* has $m_{i}^{out}=\sum_{j\neq i}m_{ij}$ outgoing mentions and $m_{i}^{in}=\sum_{j\neq i}m_{ji}$ incoming mentions. In the null model all edges in the graph are severed and outgoing edge stubs from each region are reconnected to all other regions in proportion to their original share of incoming edges. Thus region *i* has $m_{i}^{out}$ edges to re-assign. The probability to join one of these edges to region *j* is proportional to the number of incoming edges of region *j* divided by the total number of incoming edges (excluding region *i* since we avoid creating self-mentions in the null model). This means that the expected fraction of mentions from *i* to *j* is ${\bar{m}}_{ij}=m_{i}^{out}\frac{m_{j}^{in}}{M_{i}^{in}}$ where $M_{i}^{in}=\sum_{j\neq i}m_{j}^{in}$.

We compare the expected to observed in Fig 5. The main observation here is that all regions communicate more with London (and to a lesser degree Manchester) than the null model predicts. The volume of communication between other regions is therefore less than expected, however, the direction of net communication flow is preserved for all pairs but one: Norwich talks more to Nottingham, whereas the null model predicts the opposite. For each region we look at the ratio of total incoming to total outgoing mentions in Table 3. This paints a similar picture; only London has a ratio greater than one and the North-East (Newcastle) has the lowest ratio of all 9 regions.

## Inter-region sentiment

Regional identities and rivalries lead to strong emotions about sport, politics or any number of issues. Local stereotypes may lead to negative associations with a particular place. By analysing the text of the messages exchanged between regions we can ask if these expectations are reflected in the sentiment of the communication.

Sentiment analysis on Twitter is another large topic. Early work used sentiment analysis of tweets to try to predict elections, [25] movie box-office returns [26] and brand sentiment [27], demonstrating the power of the approach. Much research has been done on improving sentiment analysis for short texts like tweets or SMS messages [28–30]. We use a popular lexicon-based sentiment analyser [31, 32] to to assign a polarity to each tweet. Polarity is a number between -1 and 1 measuring the negative or positive sentiment in a text. The sentiment analyser of [32] was originally trained on a corpus of movie reviews. We have compared polarity measured by [32] to sentiment scores calculated using rule based approaches [33] and found close correlation between the two methods. Applied to our large corpora, the method of [32] is a reasonable and consistent way to measure sentiment and all reported sentiment scores are highly statistically significant.

**Fig 6:**
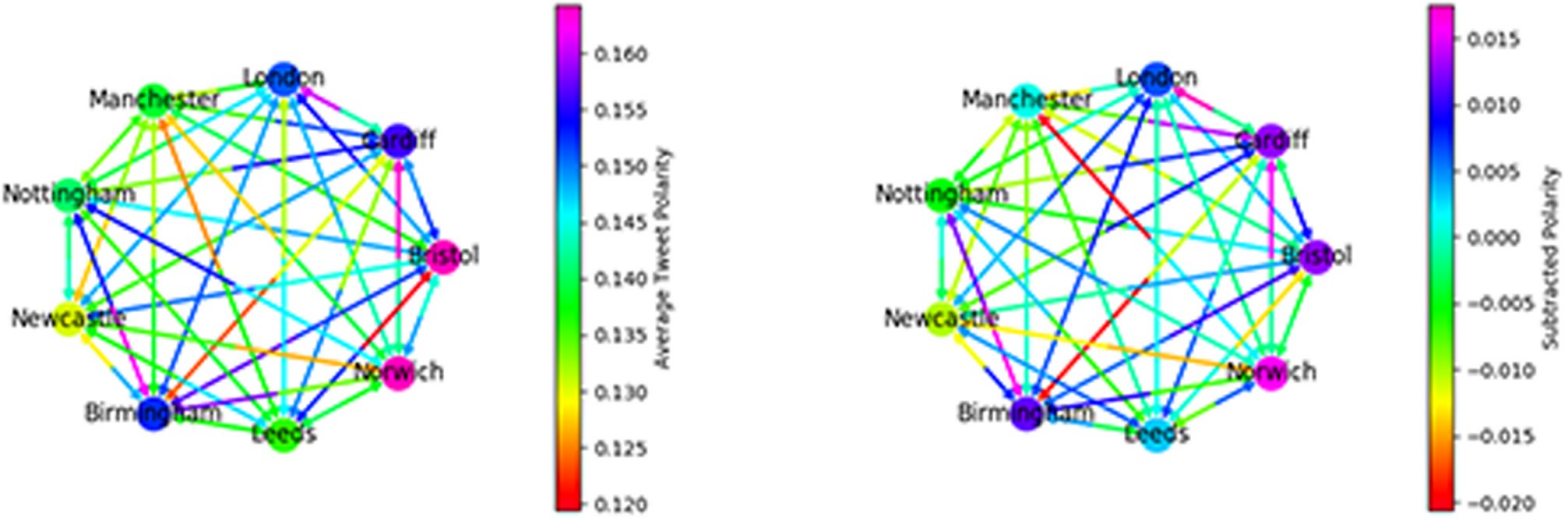
Left: Arrows: average sentiment per tweet between regions. Node: average sentiment for tweets sent within region. Right: Arrows (*p*_*ij*_ − *μ*_*i*_), nodes: (*p*_*ii*_ − *μ*_*i*_) i.e. sentiment corrected for baseline of each region.

We explore the message sentiment in two ways, again using the induced graph. Fig 6 (left) shows the average polarity of a message sent between any two communities, *p*_*ij*_. Polarity is on average positive, indicating the average tweet which mentions another user is positive. This is in line with research on sentiment in other corpora which finds a general trend towards positive polarity [34]. Self-polarity is shown as the node color, indicating that southern regions are more positive in both inter- and intra-community communication. See Fig C in S1 File for an example of the distribution of *p*_*ij*_ for one pair of communities. We estimate errors for the average sentiment score by bootstrap resampling—for each pair *ij* we resample the list of sentiment scores and calculate the resampling distribution and use this to estimate a 90% confidence interval, which we convert into a symmetric error bar below.

To determine the background level of sentiment for each region, for each community let $\mu_{i}=\frac{1}{N}\sum_{j}p_{ij}$. Each arrow or node in Fig 6 (right) shows ${\tilde{p}}_{ij}=\left(p_{ij}-\mu_{i}\right)$. As differences in regional vocabulary may lead to some regions sending tweets with lower measured polarity scores, this procedure allows us to look at inter-region communication relative to the baseline in each region. See Table B in S1 File for exact values, with errors. As we have so many mentions, most average polarity measurements are quite precise, smaller than the resolution of the colormap, the largest errors are between distant pairs of small regions e.g. Newcastle and Norwich. Fig 6 (right) shows that after correcting for background sentiment, southern regions are still relatively more positive about themselves. The ‘friendliest’ pair of regions, i.e. the pair with the highest ${\tilde{p}}_{ij}+{\tilde{p}}_{ji}$ are the neighbouring Midland regions Nottingham and Birmingham, while the least friendly are Birmingham and Cardiff. This implies spatial proximity alone does not account for inter-region sentiment.

We calculate two additional metrics based on polarity, $s_{i}=p_{ii}-\frac{1}{N-1}\sum_{i\neq j}p_{ij}$ and ${\tilde{P}}_{i}=\frac{1}{N-1}\sum_{j\neq i}{\tilde{p}}_{ji}$. *s*_*i*_ measures how positive a region is in communication with itself compared to its communication with other regions, its ‘self-regard’. ${\tilde{P}}_{i}$ measures how positive other regions are about region *i*, its ‘popularity’. Values are shown in Table 4. Southern regions have slightly positive *s*_*i*_, so they are more positive about themselves than about other regions, northern regions tend to be neutral or negative. Perhaps surprisingly, given its centrality in political discussions, London is the region with the highest incoming polarity from the other regions i.e. the most popular.

**Table 4:** Self minus outgoing sentiment, *s*_*i*_, and average incoming sentiment,${\tilde{P}}_{i}$.

Region	*s*_*i*_	Region	${\tilde{P}}_{i}$
Newcastle	-0.0116(8)	Manchester	-0.0108(3)
Nottingham	-0.0059(5)	Newcastle	-0.0049(7)
Manchester	0.0012(4)	Norwich	-0.0023(8)
Leeds	0.0036(5)	Leeds	-0.0006(4)
London	0.0072(2)	Nottingham	-0.0006(5)
Birmingham	0.0130(5)	Bristol	0.0014(4)
Cardiff	0.0145(6)	Birmingham	0.0038(4)
Bristol	0.0145(6)	Cardiff	0.0038(6)
Norwich	0.0180(11)	London	0.0042(2)

${\tilde{P}}_{i}$ which measures the sentiment of the other regions for the target region. Norwich has the most self-regard, *s*_*i*_, Newcastle the least. London is the most popular region and Manchester is the least.

## Conclusions

This paper is intended as a methodological guide as well as a case study of England and Wales. Traditional regional identities are reflected in social media interactions. Located tweets are a unique resource that allow both community identification and analysis of inter-community communication. We have examined the volume of messages sent between regions, the vocabulary/topics within a region versus the vocabulary/topics used to communicate with other regions, as well as sentiment between regions. As we have shown for England and Wales, these considerations lead to some interesting conclusions in terms of dialect, conversation topics, information flow and expressed sentiment and we predict that the same methodology could be fruitfully applied anywhere where Twitter use is high.

We identify several key results from our study of England and Wales

- Tweets from Wales are (statistically) significantly different, in terms of dialect, than tweets from England. Similarly, tweets from the North-East are significantly different than tweets from the South.
- The topics of intra- and inter-regional communication differ. Sporting rivalries and local places and events occupy the intra-regional discourse, while national politics occupies the inter-regional discourse.
- London is the most popular target for tweets but population alone does not explain communication flow (cf. Norwich and Newcastle).
- Northern regions have lower *s*_*i*_ scores (self-regard) than southern ones.

We must be cautious in our analysis and recognise that Twitter (like all surveys, solicited or not) is not giving us an unbiased view of society at large. Online data clearly does not capture the views of any people who are not online; this may be an important consideration for applications in some geographical regions. Twitter is heavily urban [17, 35] and over-represents e.g. younger, higher-income people [36]. Twitter is also a platform that is used more to discuss news and social issues than personal communication, in contrast to say LinkedIn or Facebook which have different characteristic uses. This is both a feature, allowing us potential access to contentious or divisive topics, and a bug e.g. in sentiment analysis, we could be examining an unusually negative corpus. This also has consequences for the volume of tweets—the news and politics focus of Twitter is perhaps another reason that London, seat of government, finance, as well as many news organisations, is over-represented. More broadly, this approach depends on text-mining using word frequencies, which does not include semantic information; an extension to use (e.g.) topic modelling might help to improve this aspect. Sentiment analysis, as used here, is also limited in its ability to correctly identify complex or cryptic sentiment from short-form text such as tweets.

Nevertheless, this combination of community identification with text analysis has widespread application. Marketing and political campaigns could potentially use this methodology (perhaps at a smaller scale than national) to identify relevant local issues or if they are targeting single or multiple ‘communities’, which may respond better to different messages. Beyond practical applications, this methodology has the potential to build a quantitative, econometric basis for the study of cultural exchange. The agents of this quantitative theory are the emergent regions, and we can use this combination of social-media data, network science and text analysis to shed light on regional discourse, dialect, connectivity or possibly even regional tension in an area more fractious than the UK. This method provides a way to characterise regions and both suggests interesting social questions (e.g. why does Norwich have such a large ‘influence’ relative to its population?) and also provides the empirical data to *quantitatively* test explanatory theories. In general we believe this methodology will help expose the relationship between people, social media, space and place.

## Supporting information

S1 File Robustness checks and additional tables. (PDF)
