# Supplementary material for: The human geography of Twitter: Quantifying regional identity and inter-region communication in England and Wales
Source: PLoS One. 2019 Apr 15;14(4):e0214466. doi: 10.1371/journal.pone.0214466 (PMC6464185; doi:10.1371/journal.pone.0214466)
Supplement: S1 File — (PDF) [file pone.0214466.s001.pdf]

Supporting Information

Robustness Checks and Additional Tables.

Varying the Grid Resolution

Here we study how the partition of England and Wales into regions depends on how we aggregate users. We, as in previous work [1], look at the connections between grid tiles rather than users themselves. The size of the grid tile is chosen by us, we simply divide our bounding box into an $X \times X$ grid. Given this somewhat arbitrary choice it is important to investigate the dependence on the chosen grid resolution. Grid resolution could affect our results in three ways. Firstly, our method uses the overlap between grid boxes and the spatial polygon associated with a tweet to determine which boxes the tweet should be added to, so the relative sizes of tweet polygons and grid tiles affects the likelihood of a polygon being completely contained within a tile. Secondly, the number of possible nodes and edges in the network is determined by the number of grid tiles. Third, the edge weights depend on the numbers of tweets aggregated within each grid tile. Here we compare different grid resolutions to test sensitivity of the regions that are produced.

Fig A shows three grids, Coarse (10×10), Medium (30×30, which we use in the main text) and Fine (52×52). We see that the Coarse grid identifies 5 regions (roughly: South-East, Wales & South-West, Midlands, North-East and North-West). Clustering on the Medium grid identifies the 9 regions discussed in the text. Clustering on the Fine grid identifies the same 9 regions, plus two small additional regions, centered around the cities of Stoke-on-Trent and Southampton. Modularity increases going from the Coarse (0.086) to Medium (0.209) to Fine (0.255) grid resolutions.

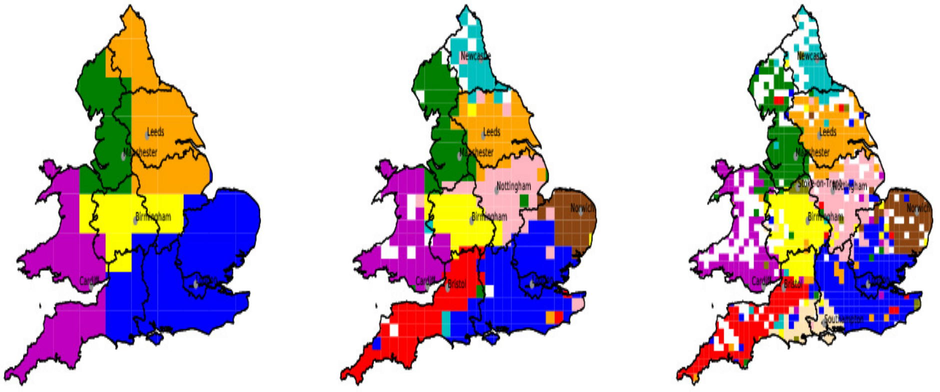

Fig A. Varying Grid Resolution. Left to right: Coarse, Medium and Fine grids.

There is no *a priori* reason to prefer one grid resolution over another. In this case, different choices allow different hierarchical levels of structure to be distinguished. Our

choice of the 30×30 grid for primary study is motivated by the observation that it is the coarsest grid that roughly reproduces the administrative regions of England and Wales. Very small communities have low volumes of communication to/from them and it becomes difficult to make statistically meaningful statements.

As we increase the resolution further we find further subdivision of the regions e.g. a small part of the South-East splits into its own community centered around Southampton. Our final choice is a compromise between high modularity and large volumes of communication data flowing between the regions. Furthermore, the same users and tweets are included in the common regions identified by the Medium or Fine grids. This means we would obtain the same results if we performed the analysis using the Fine grid, though with some edge cases differing slightly and some communities splitting at the periphery. Although the modularity is bigger for the Fine grid, these considerations lead us to choose the Medium grid in preference.

Studying Time Dependence

As another robustness check, we split our data set roughly in half. One half consists of tweets authored in 2017 the other half, tweets from 2018.

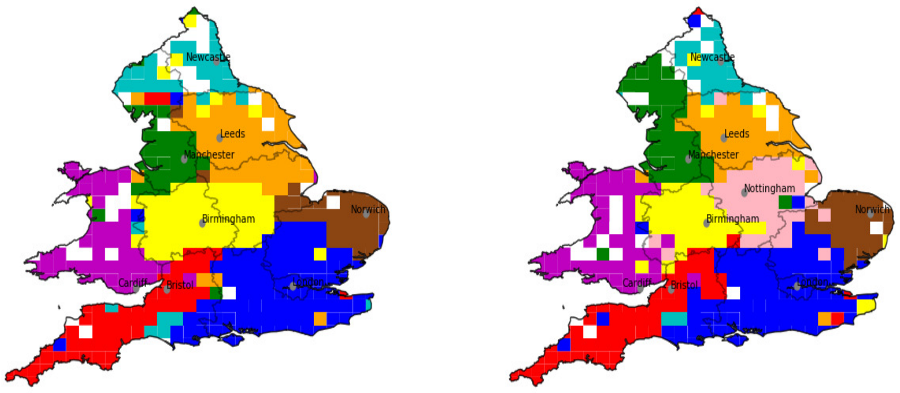

Fig B. Studying Time Dependence. Left 2017 tweets, right 2018 tweets.

Studying Fig B we see that, broadly, the same regions are reproduced using the subsampled datasets. The major anomalies are 1. a slight difference in the western extent of the Newcastle region using the 2017 data and 2. the absence of the Nottingham region on the 2017 map. Regarding 1, the extreme north-west of England is very sparsely populated and the source of very few tweets. As such, its assignment has little effect on the modularity of a partition and a slight change in the number of tweets between grid squares can change the optimal assignment of these tiles. This is another manifestation of the fact that very sparsely populated areas, as the source of few tweets, are more difficult to assign to a community.

The second anomaly is the absence of Nottingham. As we have shown above, on our coarse grid, communities can split and join depending on the resolution we are using for our study. This result implies that Nottingham is not as strongly distinct from Brimingham and Leeds as e.g. Norwich and that more data is necessary for this community to be detectable. Effects like transient populations of students/tourists or large news/cultural events could account for the 'appearance' of Nottingham in 2018. However we find it plausible that with less data, some communities may be statistically

indistinguishable, as is the case for any clustering problem. The issue of time dependence is interesting and worthy of future study, however for this manuscript we will work with our entire dataset and use the 9 communities found from this analysis, including Nottingham.

Text A. Top 100 'Local' Terms by TF-IDF.

London

trndnl mymyselfandmyphotos afirmremain kenonmkfm greenwichhour ealinghour womened jbombs catford freelancephotographer artconnects drivingforward mcmlvp cosplaygirls countryradio automotiveindustry rbemusicshowcase sneakerjeans mcmcomiccon ldnmcmcomiccon sqsc partyclubmix mcmmidlands willink filteredphotos britishsports forkster noskillphotography cosplayer lewisham controlyourbody southsea farmoor tooting gohartsc wearelondon headington brownout gistwithcynthia commercialvehicle leighonsea cheam hertshour govia iicsa eyesofladyw trumpdossier mylocalculture ukbtsarmy toxicsurv upthehamlet wristinstability cosplaymasquerade lovemk removerutnamskcb southeastern mictizzle ncsupperclubs newham bwsfb ldnmovesme visitbanbury upthestoke muckymerton feedthepoor meaow coycards cobbett cultureofukafrobeats boweldisease wearerugby londonfashionweek thelindaeshow samplesale osteopathy midcarpalinstability dhfc elft runwithandy bostikpremier gamificationeurope ulcerativecolitis northlondonhour otmoor mertonculture southlondon londres lovemycity ifgs herberts digme moveitshow axschat wristpain wandsworth successhour rbwm ruinadatein engagors beckenham

Manchester

thefootball oldhamhour marktokerrys beeryview cheerstobeers adelaidies ffrindiau evostikleague lbe leadinggm urmston tweacon tameside camrgb oneport aufc sthelenshour eclcm savemajorcrimes calderstones bolllocks chestertweets corinne itsliverpool cfba mdso fabcan saletown poggy newbrew stav kirbs larklane cumbrianbeer isplenty churchgate theys kerrys prestonhour ourmanchester bevys faithinyoungpeople ancoats ministryofsport knitswithbeer jukeboxthursdays manchestercentralfc swanfest gmcuk purpleandproud janeballlandscapes wxmafc codarmy gan prosandcoms pieceofrubbish oafc loveforever upthetics prelovedhour chorlton majorcrimes lancashirehour sthelens wallasey fmcreators thinkfaster wythenshawe bestshowontv teamukfast onesalefc timperley proudtofoster proudtoadopt uhn mancmade teamorrell janeballphotography raggies wwoolfall altrincham indiehour mcrchristmas ourtownsteam rra hulme piccadillyward lunchtimers dazzzzer chorley cwfl moston poggys hogblog slingthemesh pnefc gbar fitnessisakeyrequirement baltictriangle refperformance

Bristol

niks cornwallgate exeterhour bathindiechat swindonhour ukcachehour cmonbris mojorocks communityownership somersethour cornishhake exeterlive freshcornish countrykids devourbrischat champrugby nerdschatting remoanersbs mikevigorfanclub ourchelt dailyinvinciblecover wnukrt theendofallthings glawsfamily devonhour englishriviera makingbristolproud savebathfromboring crediton daretodisrupt selworthy smwbristol musichouruk philskillerthree glosbiz barnstaple ericalovesyou packthegate torbayhour winchcombe torbay merrybrexmas lovethebarbican bristolrugby womaninbiz teahour jacki officialgettogether newlyn walt nsomerset exmoor finzelsreach tiverton cind glaws hagx bewhatyouare cockington swtawards

cornishfootball ukwinehour signedupsaturday remainfakenews bris futurecity saltash babbacombe capetown devonfoodhour wickedontour nbtproud babber opmfamily amazingarchie cwlbirds treesofthisworld paignton teignmouth glos boosttorbay brivbed maymedia cornishlove remainlies veawards hetourism bristolandproud truro gloshour falmouth poyf propertyladder newtonabbothour eatmorehake transhealth amateurradio bristolbizhour topsham dartmoor

Leeds

euers yesladhull kirklate sheffieldissuper hcafc wharfedalelinks ukcolumn safclive htafc kirklatemarket bcafc euer gmy gewgo doncasterisgreat lovinleeds bigupbradford weareyork cookridge dearne connectingthepieces northstarchat hullhour theworldiswatching maboxing headingley realaleawaydays tvaddict dts ofosheffield hazlegreaves utm marketingweek standwithduchess ukwinehour backinghuddersfield sheffbeerweek ehab calumshullcrew topoftown fanthursday getskyhome nyp leedshour yesladlogo brexitnow syp dazl fido teamdylan otweeklds ijw gayleeds barnsleyisbrill nomorefelling filey taddy teamtheo scc allam projekt horsforth nostell halfhourofpower torbayhour gafc isdead stophs amey geofleurshop bdxmas dewsbury saveshefftrees teambentsgreen dwba wahaw ramember selloutsaturday ataw ryuvoelkel liff teambradford meersbrook sheffieldhour allams geddes hkr burnsy goc menston calderdale roundhay cits fearthefin midstaff supportlocalmarkets enjoyyourprivilege abilitiesnotdisabilities teamyas brighouse

Cardiff

yn introbiz dwi oneclubonecounty introbizexpo herefordgoals usw ddim wedi jdwpl valueadded betws iawn nawr roath edrych llongyfarchiadau ukdiscoversaudi chdi ymlaen efo oedd alfieandwilf heddiw sportcardiff mewn ydi hynny rbcdn jofli heno redbalwncoch ytad letsgodevils yna hefyd cael llanelli ond gyfer savemajorcrimes mwynhau uswrugby uptheport daysofselfcare bawb ffordd theglovesareon meddwl caerdydd murenger fel wruin wrth aberdare pubofdreams ndwg wneud pigparker heretoplay siarad penarth gyda gyd poorparkingcdf hellocolin hefo neud bigtuesdayshow uhw erbyn gymraeg rwan fynd ringland ysgol depressionrecovery llanedeyrn rhondda rhaglen cardiffmet llanishen newydd bethespark chwarae angen vitallis llandaff ystrad wyburn wearredforwalesandvelindre cathays sydd virtualworldrideforrity ammanvalley sesiwn siwr grangetown gweld roedd

Norwich

norfolkhour skullswasps livinghistory allezallezallez norwichhour euroland theveryear suffolkhour upthepeckers holtbirdcafe norfolkfootball felixstowe alycia yarmouth uea tryyy autisticandproud autismaware norvwas wickeduk charitygala bigdaddyfamily badtouch rolloff facoachmentor liveworklovenorfolk garboldisham burystedmunds wearewsft drinkextraordinary northnorfolkcoast fidel caw marketmen cromer vhurrell oldchimneysbrewery nmfc erwfl holkham soyuztour akingsransom planetsuffolk weareabode wasvlei tryyyy autisticsinger promnation proudcanariesfc edpphotographer lavenham pltv forecastingchallenge grandmentors pdnreflect canarycall ueailm lovethekick communitypub gomagpies dereham beccles mfta swanlavenham commitioner srbeny norwichpubs wasvhar klose pricklypals greatyarmouth teamnnuh fawomenscup itlfc pricklypal tayfen cihcareersweek otbc fawpl woodbridge hotelschool teamwicked emwfl kyley plasterersarms sudbury thetford harvwas needhammarketfc kingslynn stowmarket hoglet sundayatthemusicals holthogcafe trowse

norfolkrestaurantweek buildthenest norwichlighttunnel norwichlights foxythanks

Birmingham

covhour morphettes thisiscoventry brumhour worcestershirehour jewelleryquarter covhourlive leamingtonhour sociallyshared noflyzone kingsheath xhx lovebrum suahour bizw ipex cwrocks bilbrook thepaguide lovelydrop eastsidejazzclub nearbywild digbeth tuffnut pusb kfe progmill sutcolhour pedsicu thekindnessofpeople salop newbuckshead harrisgibbshair dayswild jonworcesterman willhomewoodbirmingham afrin soulstew secondsinbrum warwickshire tonetaxi covcab grumpys teamwolves cwbf atruegent loveleam harborne biggen gbccexpo nowplaying yeworcestershire blazemotm rockly yecompany studley wearebeingwarned paintwithcars mtptproject haircolour silvermountainagency dosanjh carlingo wehavebeenwarned brumbloggers wolvesaywe stirchley bypy moleend lovesolihull chamberlinkdaily bcccawards hellobrum whitneyout nuno tasteofsrilanka olton pyfproms ckob blackcountry malvernhills jq birminghamartists bonser bubblebobauk lifeecho fosun bridgnorth huis mijn zyderney getinvolvedbrum thriveawards glassboys pelsall brumindependents brumtechawards fwaw boldmere malvernhillshour

Nottingham

lollol nccc wherehistorybegins fankew blatherwick nonlge doigy dilnot pineappleoregg whowzers dogtanian lbororugby cousion betteridge cousions jaqui luking gudvkuking boppy simmos bforbhour gasteric byepass changingbeers jannet teamdmu blatherwicks proo earlycrew panthersnation tkofficialmc sethslegacy lborowalksonwater doigys hauntedthursday askuon psyed bennifit includeing footbsllers rebete agentr disolsys lborofamily thff picheal teambeswicks nantwich lboro earings pvfc trentham westerne lovestoke pancanstory lwow southerton wearelincoln votecomedy jsnnet stokeontrent faydeesupporter greennwhites wlechat teamngh armyred boppys farndale proudtobestaffs thrapston nffcclub faceofsot bfbd farndales twitterposh ripleys faydeearmy hcfc antoney greenandgold dilnots djfestlei nearley marksfacuptour loveintheafternoon togetherforautism leicscomedyfest myk globelist tennereefe valproate fatrophyquest theverseandguests lanzorottee ukmodel jasondenoshow kenty laughterloft welovenewhomes fingertoescrossed

Newcastle

votecategories consett nefollowers tnarmy prayfortidal leadgate voterfraud coymmp pelaw sparkysrunningclub sundayya totalsport votingcountmodel cheryll northeasthour morekidsonbikes enlscores goffy mancrushforever thisismine northeast corbynistalife northeastcreatives newcastlescaleupsummit letsgoeagles newcastlegateshead veteranslivesmatter utb howayblyth stillflowering hawaythescholars cillamoment templeofboom morpeth getnorth epdp uptheglebe digitalshowcase ultravires boldon hebburn metrocentre cdsou teesvalley teesside chesterlestreet stopthehate welcometosunderland southshields allhailtotheale tidesoflove turfeoke justwrestle amandain madeinnewcastle thisnortherngirlcan edinburghisblackandwhite gateshead hitthebar shinethelightbrightaootherscansee ouseburn aycliffe cramlington tvbcmembers robmcavoy derwentside sackrodwell narrowmind heworth stockton mackem votercountmodel seaham whitleybay uofefutsal cakelicious nonpolitical intothewoods nebloggers boostyourbusiness blobbys babyitschristmas trfc kone bewateraware jesmond teammcjonesforthewin sunderlandfutures boty secundus uptheesh mushroomworks weekendanthems nulli

inrafawetrust longbenton roker teammcjones newcastleupontyne haway

London	Manchester	Bristol	Leeds	Cardiff
liverpool(613)	brexit(694)	labour(783)	brexit(682)	brexit(709)
sleep(369)	government(612)	brexit(603)	eu(594)	eu(653)
trending(337)	tory(572)	nik(568)	leaving(570)	series(580)
ff(316)	nhs(506)	retweet(500)	nhs(539)	nhs(568)
topic(304)	eu(493)	eu(467)	ref(520)	labour(537)
co(-396)	wigan(-475)	lunch(-596)	pop(-497)	ar(-564)
brighton(-423)	chester(-504)	breakfast(-617)	south(-504)	coach(-570))
lunch(-449)	lunch(-515)	swindon(-654)	thursday(-512)	photos(-582)
awards(-472)	gt(-564)	cheltenham(-656)	council(-564)	project(-597)
greater(-818)	mufc(-606)	council(-716)	bradford(-586)	iawn(-852)

Norwich	Birmingham	Nottingham	Newcastle
song(597)	brexit(803)	eu(787)	country(567)
hell(533)	disabled(663)	brexit(696)	mum(558)
cannot(507)	extremely(654)	labour(568)	bro(548)
film(481)	inclusion(654)	nhs(442)	vote(538)
answer(461)	wwfc(518)	tour(437)	sweet(508)
students(-515)	thursday(-491)	dave(-509)	nowt(-502)
latest(-517)	pigeonswoop(-504)	lincoln(-520)	boro(-585)
involved(-522)	villa(-557)	steve(-530)	students(-656)
ncfc(-529)	art(-692)	council(-542)	gateshead(-677)
lunch(-664)	blues(-700)	aswell(-881)	safc(-778)

Table A. Rank Differences. Top and bottom five rank differences for all regions.

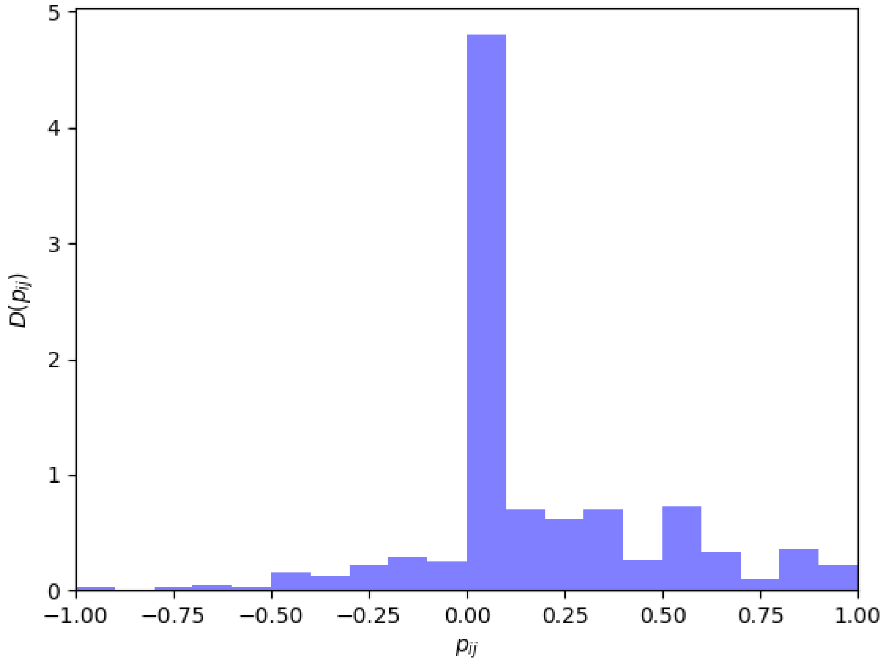

Fig C. Sentiment Distribution. Example of the distribution of sentiment scores for mentions going from Bristol to Cardiff. The large peak at 0 is due to the fact that many tweets do not have an measurable sentiment. The other distributions are very similar in appearance. See the main text for details on estimating errors and confidence intervals for p_{ij}

Region i	Region j	p_{ij}
Bristol	Bristol	0.164(1)
Bristol	Cardiff	0.149(2)
Bristol	London	0.154(1)
Bristol	Manchester	0.140(1)
Bristol	Nottingham	0.146(2)
Bristol	Newcastle	0.150(3)
Bristol	Birmingham	0.157(2)
Bristol	Leeds	0.152(2)
Bristol	Norwich	0.149(4)

Region i	Region j	p_{ij}
Cardiff	Bristol	0.152(2)
Cardiff	Cardiff	0.156(1)
Cardiff	London	0.161(1)
Cardiff	Manchester	0.136(1)
Cardiff	Nottingham	0.134(3)
Cardiff	Newcastle	0.136(5)
Cardiff	Birmingham	0.124(2)
Cardiff	Leeds	0.150(3)
Cardiff	Norwich	0.142(6)

Region i	Region j	p_{ij}
London	Bristol	0.148(1)
London	Cardiff	0.143(1)
London	London	0.151(0)
London	Manchester	0.132(1)
London	Nottingham	0.140(1)
London	Newcastle	0.148(1)
London	Birmingham	0.151(1)
London	Leeds	0.146(1)
London	Norwich	0.143(2)

Region i	Region j	p_{ij}
Manchester	Bristol	0.137(1)
Manchester	Cardiff	0.152(2)
Manchester	London	0.138(0)
Manchester	Manchester	0.139(0)
Manchester	Nottingham	0.133(2)
Manchester	Newcastle	0.127(2)
Manchester	Birmingham	0.138(1)
Manchester	Leeds	0.137(1)
Manchester	Norwich	0.140(3)

Region i	Region j	p_{ij}
Nottingham	Bristol	0.148(2)
Nottingham	Cardiff	0.154(3)
Nottingham	London	0.146(1)
Nottingham	Manchester	0.135(1)
Nottingham	Nottingham	0.141(1)
Nottingham	Newcastle	0.143(3)
Nottingham	Birmingham	0.162(1)
Nottingham	Leeds	0.139(1)
Nottingham	Norwich	0.146(4)

Region i	Region j	p_{ij}
Newcastle	Bristol	0.145(3)
Newcastle	Cardiff	0.148(4)
Newcastle	London	0.147(1)
Newcastle	Manchester	0.133(2)
Newcastle	Nottingham	0.141(3)
Newcastle	Newcastle	0.130(1)
Newcastle	Birmingham	0.148(3)
Newcastle	Leeds	0.147(2)
Newcastle	Norwich	0.126(7)

Region i	Region j	p_{ij}
Birmingham	Bristol	0.152(2)
Birmingham	Cardiff	0.130(2)
Birmingham	London	0.149(1)
Birmingham	Manchester	0.132(1)
Birmingham	Nottingham	0.153(2)
Birmingham	Newcastle	0.129(3)
Birmingham	Birmingham	0.152(1)
Birmingham	Leeds	0.138(2)
Birmingham	Norwich	0.134(4)

Region i	Region j	p_{ij}
Leeds	Bristol	0.119(2)
Leeds	Cardiff	0.133(3)
Leeds	London	0.132(1)
Leeds	Manchester	0.125(1)
Leeds	Nottingham	0.135(2)
Leeds	Newcastle	0.137(2)
Leeds	Birmingham	0.137(2)
Leeds	Leeds	0.136(1)
Leeds	Norwich	0.139(4)

Region i	Region j	p_{ij}
Norwich	Bristol	0.144(4)
Norwich	Cardiff	0.164(6)
Norwich	London	0.148(1)
Norwich	Manchester	0.128(3)
Norwich	Nottingham	0.153(5)
Norwich	Newcastle	0.136(7)
Norwich	Birmingham	0.158(4)
Norwich	Leeds	0.140(4)
Norwich	Norwich	0.164(1)

Table B. Polarities. Average polarity of mentions sent from region i to region j. Number in bracket is error on last digit.

References

1. Ratti C, Sobolevsky S, Calabrese F, Andris C, Reades J, Martino M, Claxton R, Strogatz SH. Redrawing the Map of Great Britain from a Network of Human Interactions. PLoS ONE. 2010;5(12):e14248.
